# Synergism of FAK and ROS1 inhibitors in the treatment of *CDH1*-deficient cancers mediated by FAK-YAP signaling

**DOI:** 10.7150/ijbs.81918

**Published:** 2023-05-15

**Authors:** Jiaming Gao, Yunying Yao, Chenxuan Liu, Xi Xie, Donghe Li, Ping Liu, Zaiqi Wang, Baoyuan Zhang, Ruibao Ren

**Affiliations:** 1 Shanghai Institute of Hematology, State Key Laboratory for Medical Genomics, National Research Center for Translational Medicine at Shanghai, International Center for Aging and Cancer, Collaborative Innovation Center of Hematology, Ruijin Hospital affiliated to Shanghai Jiao Tong University School of Medicine, Shanghai, China.; 2 International Center for Aging and Cancer, Hainan Medical University, Haikou, Hainan Province, China.; 3 InxMed (Shanghai) Co., Ltd, Shanghai, China.

**Keywords:** FAK Inhibitor, ROS1 inhibitor, Combination therapy, drug resistance, YAP-TRX axis

## Abstract

*CDH1* deficiency is common in diffuse gastric cancer and triple negative breast cancer patients, both of which still lack effective therapeutics. ROS1 inhibition results in synthetic lethality in *CDH1*-deficient cancers, but often leads to adaptive resistance. Here, we demonstrate that upregulation of the FAK activity accompanies the emergence of resistance to ROS1 inhibitor therapy in gastric and breast *CDH1*-deficient cancers. FAK inhibition, either by FAK inhibitors or by knocking down its expression, resulted in higher cytotoxicity potency of the ROS1 inhibitor in *CDH1*-deficient cancer cell lines. Co-treatment of mice with the FAK inhibitor and ROS1 inhibitors also showed synergistic effects against *CDH1*-deficient cancers. Mechanistically, ROS1 inhibitors induce the FAK-YAP-TRX signaling, decreasing oxidative stress-related DNA damage and consequently reducing their anti-cancer effects. The FAK inhibitor suppresses the aberrant FAK-YAP-TRX signaling, reinforcing ROS1 inhibitor's cytotoxicity towards cancer cells. These findings support the use of FAK and ROS1 inhibitors as a combination therapeutic strategy in *CDH1*-deficient triple negative breast cancer and diffuse gastric cancer patients.

## Introduction

Deficiency for E-cadherin protein caused by mutations in the cadherin 1 (*CDH1*) gene is a major driver of tumorigenesis in diffuse gastric cancer (DGC) and in breast cancer 1, 2. Patients with *CDH1* deficiency generally show the worst prognosis and shortest overall survival time in DGC and triple-negative breast cancer (TNBC) 3, 4. Due to the lack of effective targeted therapies, conventional chemotherapy continues to serve as the standard of care for treating DGC and TNBC. However, clinical outcomes of conventional chemotherapy are typically poor, with substantial toxicity mitigating further benefits of treatment 5, 6. Hence, novel regimens targeting *CDH1* deficiency are urgently needed.

Targeting the receptor tyrosine kinase, ROS proto-oncogene 1 (ROS1), can lead to synthetic lethality in cancers associated with *CDH1* deficiency 7, and several ROS1 inhibitors have been approved to treat non-small cell lung cancers by the US Food and Drug Administration (US FDA) due to their strong clinical responses 8, 9. In particular, clinical trials of the ROS1 inhibitors crizotinib and entrectinib continue in *CDH1*-deficient breast cancer and DGC patients 10, 11. However, the development of resistance to ROS1 inhibitors remains an urgent focus of clinical research, since the only remaining clinical interventions for ROS1 inhibitor-resistant cancers are chemotherapy or locally ablative therapy 12. Notably, rational combination strategies incorporating ROS1 inhibitors with a non-overlapping mechanism may facilitate some degree of success in overcoming drug resistance 13.

A growing body of evidence supports a close association between the effects of *CDH1* deficiency and focal adhesion kinase (FAK) signaling. In DGC and TNBC patients, FAK significantly promote self-renewing cancer stem cells and correlates with malignant potential 14-16. Moreover, FAK inhibitors have been shown to attenuate tumor growth in *Cdh1*-deficient mice and show potent cytotoxic effects in DGC and TNBC cell lines 17, 18. FAK also functions as an informative biomarker of cellular stress and plays a key role in promoting resistance to chemotherapy and various targeted therapies 19. Co-treatment with FAK inhibitors, including inhibitors of the B-Raf proto-oncogene (BRAF), mitogen-activated protein kinase (MEK), and KRAS-G12C, is currently well-accepted as a viable potential strategy for overcoming adaptive resistance to chemotherapy, radiotherapy, or targeted therapies 20-24, supported by evidence emerging from a series of clinical trials evaluating FAK inhibitor combination regimens 25. In light of these findings, we hypothesized that ROS1 inhibitors used in combination with FAK inhibitors could provide durable benefits for DGC and TNBC patients with *CDH1* deficiency.

In the present study, we tested this hypothesis and found that co-treatment with ROS1 inhibitors and FAK inhibitors conferred synergistic anti-tumor effects in *CDH1*-deficient mice model of DGC *in vitro* and *in vivo*. Additional experiments indicated that FAK-YAP-thioredoxin (TRX) signaling was enhanced by administration of a ROS1 inhibitor, sequentially decreasing the oxidative stress-related DNA damage, which compromised the effects of the ROS1 inhibitor. Treatment with the FAK inhibitor, IN10018, which has been granted fast-track designation by the US FDA, led to enhanced effects of ROS1 inhibition by rescuing aberrant FAK-YAP-TRX signaling. Since there are currently no highly selective ROS1 inhibitors available (*e.g.*, crizotinib targets ROS1, anaplastic lymphoma kinase (ALK) and mesenchymal epithelial transition factor receptor (MET) kinase), we also investigated whether the observed anti-tumor effects were indeed achieved through ROS1 inhibition. Silencing of each predicted target in NUGC-4 cells showed that ROS1, but not other targets, was critical for the synergistic effects of combination therapy with IN10018. These findings suggest that co-administration of FAK inhibitors and ROS1 inhibitors may be an effective potential treatment option for *CDH1*-deficient cancer patients and warrant further testing in clinical trials.

## Materials and Methods

### Reagents

Crizotinib, entrectinib, and VS-4718 were purchased from MedChemExpress (MCE). IN10018 was provided by InxMed. All the siRNAs used in the study were synthesized by Genepharma (siRNA sequences given in Table S1 of the Supplementary materials). The antibodies used in the study are listed in Table S2 of the Supplementary materials.

### Cell Culture

DGC cell lines were cultured in RPMI-1640 medium with 10% fetal calf serum and 1% penicillin-streptomycin (Life Technologies). MDA-MB-231 cells were cultured in DMEM supplemented with 10% fetal calf serum and 1% penicillin/streptomycin (Life Technologies). Hs-578t cells were cultured in DMEM supplemented with 10% fetal calf serum, 0.01 mg/mL insulin, and 1% penicillin/streptomycin (Life Technologies). The crizotinib-resistant cell line was continuously cultured with crizotinib at a concentration that increased stepwise for 6 months.

### Cell Viability Assay

All cells were seeded in 96-well plates at a density of 3,000 cells per well. After cells were cultured for 12 h, drugs were added to the medium. Following treatment for 72 h, cell viability was determined using the CellTiter-Glo luminescent assay (Promega) according to the manufacturer's protocol. Viability was detected by a microplate plate reader (Thermo Fisher Scientific). Dose-response curves were generated using GraphPad Prism.

### Cell Clonogenic Assay

All cells were seeded in 12-well plates at a density of 1,000 cells per well. After cells were cultured for 24 h, drugs were added to the medium. The cells were maintained for 2 weeks with drug treatments. Colonies were fixed at 4% paraformaldehyde (Sigma-Aldrich) for 15 min and stained with crystal violet (0.5% w/v, Sigma-Aldrich) for the collection of images.

### Western Blotting

Cells were lysed with RIPA buffer containing 1% protease/phosphatase inhibitors (Roche), and protein levels were quantified using the Pierce™ Rapid Gold BCA Protein Assay Kit (Thermo Fisher Scientific). Proteins in lysates were separated using sodium dodecyl sulfate-polyacrylamide gel electrophoresis and then transferred to nitrocellulose membranes (0.2 μM, Amersham Protran). Blocking and antibody incubation were performed using 5% non-fat powdered milk, followed by washing in Tris-buffered saline with 0.1% Tween 20 (TBS-T). Primary antibodies were incubated overnight at 4°C and secondary antibodies (Cell Signaling Technology) were incubated at room temperature for 1 h.

### *In Vivo* Efficacy

All experiments were approved by the Ethics Committee of Shanghai Jiao Tong University School of Medicine, and all animal studies were performed following AAALAC guidance. Balb/c nude mice and NOD scid mice were purchased from GemPharmatech Co., Ltd. and maintained under 14-h light/10-h dark cycles (dark 20:00-6:00). The use of the SNU-668 and NUGC-4 cell line-derived xenografts was approved by the Institutional Animal Care and Use Committee (IACUC) of Shanghai WuXi AppTec. Experiments involving DGC cancer PDX models 051009 and 0501013 were approved by IACUC of Nanjing Personal Oncology Biotechnology. Mice developed tumors reaching a volume of 100-200 mm^3^. Six mice in each group received oral administration once per day of one of the following drug regimens: (1) control vehicle, 25 mg/kg (0.5% Natrosol 250 HX in distilled water); (2) IN10018, 25 mg/kg; (3) crizotinib or entrectinib, 25 mg/kg; or (4) IN10018 plus (S)-crizotinib, 25 mg/kg. Tumor volumes were measured by caliper and calculated. At the appropriate end point, mice were humanely killed, and NUGC-4 tumors were harvested for Western blot analysis.

### RNA-Seq

Total RNA was extracted following the instructions accompanying the TRIzol reagent (Thermo Scientific). DNA libraries were prepared from samples by using the NEBNext Ultra Directional RNA Library Prep Kit for Illumina (New England Biolabs). Transcriptome RNA sequencing (RNA-Seq) was performed using Illumina high-throughput RNA sequencing (HiSeq 2500 sequencer). Differential expression analysis of RNA-Seq data for any two groups was performed using DESeq2 26. Genes with Benjamini-Hochberg-adjusted P values <0.05 and absolute log_2_ fold changes >1 were considered to be differentially expressed. Gene Ontology (GO) and pathway enrichment analysis using Kyoto Encyclopedia of Genes and Genomes (KEGG) for the differentially expressed genes were performed using the R package clusterProfiler, with a threshold value of P < 0.05 27, 28. Data were visualized using the ggplot2 R package (https://ggplot2.tidyverse.org/).

### Detection of ROS

Cells were co-cultured with diacetyldichlorofluorescein diacetate (DCFH-DA; Abbkine, KTB1910) at a final DCFH-DA concentration of 10 mM for 30 min. The intracellular levels of ROS were analyzed by flow cytometry.

### Comet assay

The assay was performed using the Comet Assay Kit from Abcam (ab238544) as described by the manufacturer. Briefly, cells in RPMI medium were mixed with comet agarose 1:10 (v/v) and immediately pipetted onto agarose pre-coated slides. The slides were transferred to a lysis buffer and then to an alkaline solution for 30 mins at 4ºC in the dark. The slides were analyzed by electrophoresis. The cells were stained with Vista Green DNA Dye and observed using a fluorescence microscope with an image analysis system.

### Immunofluorescence Assay and Immunohistochemistry

Cells were fixed in 4% paraformaldehyde for 20 min, and then washed four times with phosphate-buffered saline (PBS). Cells were incubated with blocking buffer (0.1% Triton X-100 and 5% bovine serum albumin in Tris-buffered saline [TBS]) for 2 h and then washed three times with PBS containing 0.1% Tween 20 (Sigma-Aldrich). Cells were incubated with the first antibody overnight at 4°C and then incubated for 1 h with fluorescent-conjugated secondary antibodies. After being washed three times for 5 min each time with PBS containing 0.1% Tween 20, the cells were observed using a laser scanning confocal microscope (Lecia Microsystems TCS 7300).

The Immunohistochemistry was performed as described 24. The primary Abs included p-FAK (1:300), YAP (1:500), γH2AX (1:400), and Cleaved Caspase 3 (1:300). Then, DAB Substrate Kit (abcam, ab64238) was used to detect immunoactivity. Hematoxylin was used for nuclear staining.

### RNA Extraction and Quantitative real-time PCR analysis

Total RNA of NUGC-4 cell samples was isolated by RNA iso plus reagent (Takara). PrimeScript RT Master Mix Kit (Takara, RR036A) was used to synthesize complementary DNA (cDNA) from 1μg RNA. In addition, quantitative PCR (qPCR) was conducted by the SYBR-green super mix kit (Bio-Rad) and the Quantstudio 7 Flex Real-Time PCR system 12 (Thermo Scientific, MA, USA). Relative expression levels were calculated by the 2-ΔΔCT method. For the primers sequences which used in the experiment were listed in Table S2.

### Statistical analysis

GraphPad Prism, version 5.0 (GraphPad Software), was used for all data analysis. Unpaired student's two-tailed t-tests were used to assess the differences between samples. Data are shown as the mean ± SEM. Statistically significant differences of p < 0.05, p < 0.01 and p < 0.001 are noted with *, **, and ***, respectively.

## Results

### FAK signaling is upregulated during resistance to ROS1 inhibitor therapies in *CDH1*-deficient cancer cells

**Figure 1:**
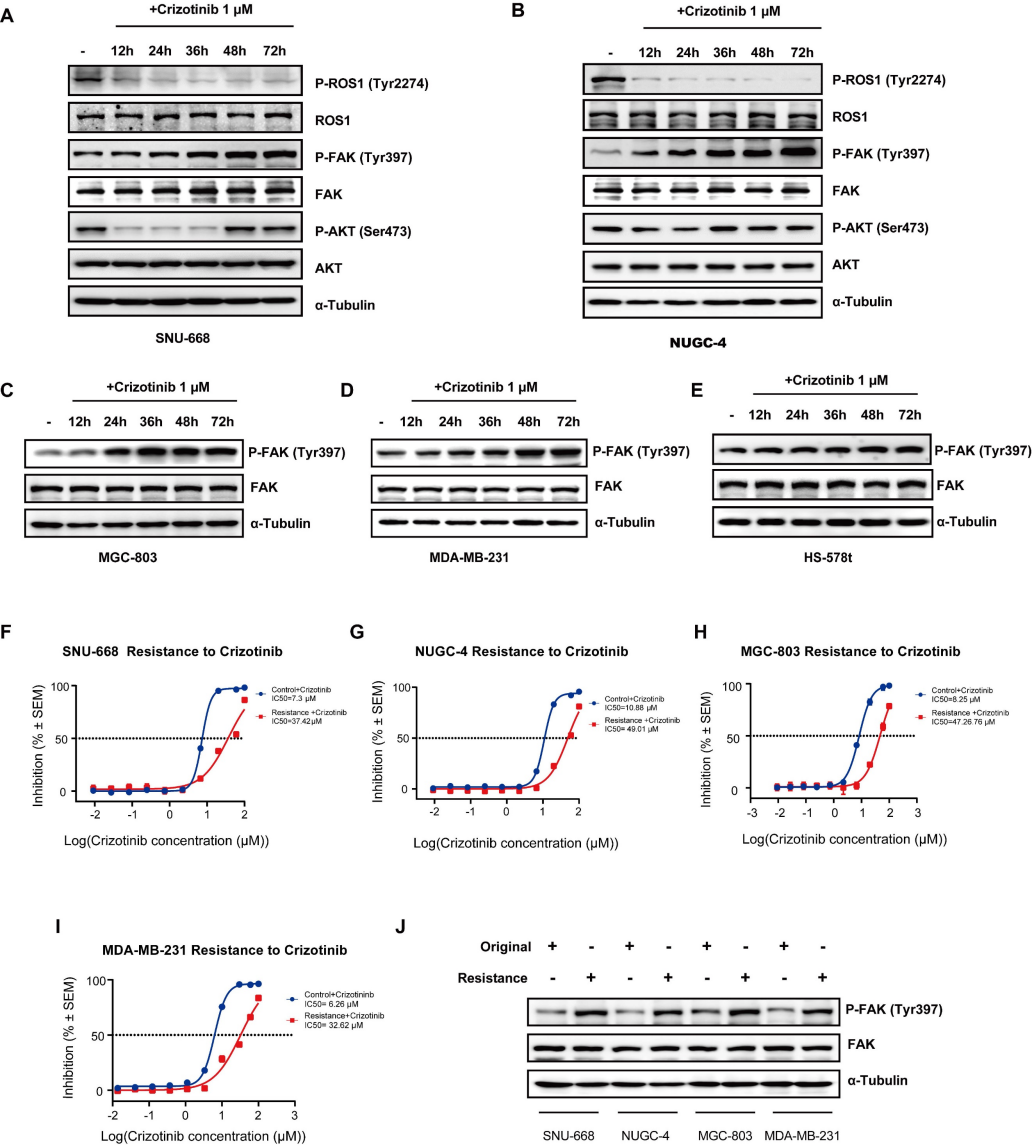
** ROS1 inhibitors increase FAK signaling in *CDH1*-deficient cancer cell lines.** (A-E) FAK signaling and downstream marker expression levels assessed by Western blot analysis at various times after crizotinib treatment. (F-I) Original and crizotinib-resistant cancer cells were treated with various concentrations of crizotinib for 72 h and assessed for viability by using the CellTiter-Glo luminescent assay. (J) Western blot analysis assessing FAK signaling in original and crizotinib-resistant cell lines.

In order to investigate whether ROS1 inhibitors affect FAK signaling to overcome drug resistance in *CDH1*-deficiency cancers, such as gastric and breast cancers, we first used Western blot analysis to determine ROS1 and FAK signaling activity levels by examining phosphorylation of their downstream targets in five cancer cell lines (SNU-668, NUGC-4, MGC-803, MDA-MB-231, and Hs-578t) across 12 hours of treatment with crizotinib/entrectinib. We found that levels of p-ROS1 significantly decreased, while p-AKT levels remained stable and p-FAK levels markedly increased, during 24 hours of treatment with either crizotinib (Figure 1 A-B) or entrectinib (Figure S1 A-B) in SNU-668 and NUGC-4 DGC cell lines. The p-FAK levels also increased with treatment time in MGC-803 cell, and TNBC cell lines MDA-MB-231, and Hs-578t (Figure 1 C-E). To determine if this observed activation of p-FAK was associated with crizotinib resistance, we established drug-resistant cell lines derived from SNU-668, NUGC-4, MGC-803, and MDA-MB-231 cells by continuous exposure to crizotinib for 6 months. A resistant phenotype was confirmed by comparing the cell killing effects of crizotinib between the original and derived cells (Figure 1 F-I). Subsequent Western blot analysis showed that p-FAK Y397 levels appeared higher in the resistant cell lines than in the original cell lines (Figure 1 J). These findings confirmed that the upregulation of FAK activity accompanies the emergence of resistance to ROS1 inhibitor therapies in gastric and breast *CDH1*-deficient cancers.

### FAK inhibition plus ROS1 inhibitors combination treatment synergistically inhibits growth of *CDH1*-deficient cancer cells with or without crizotinib resistance

**Figure 2:**
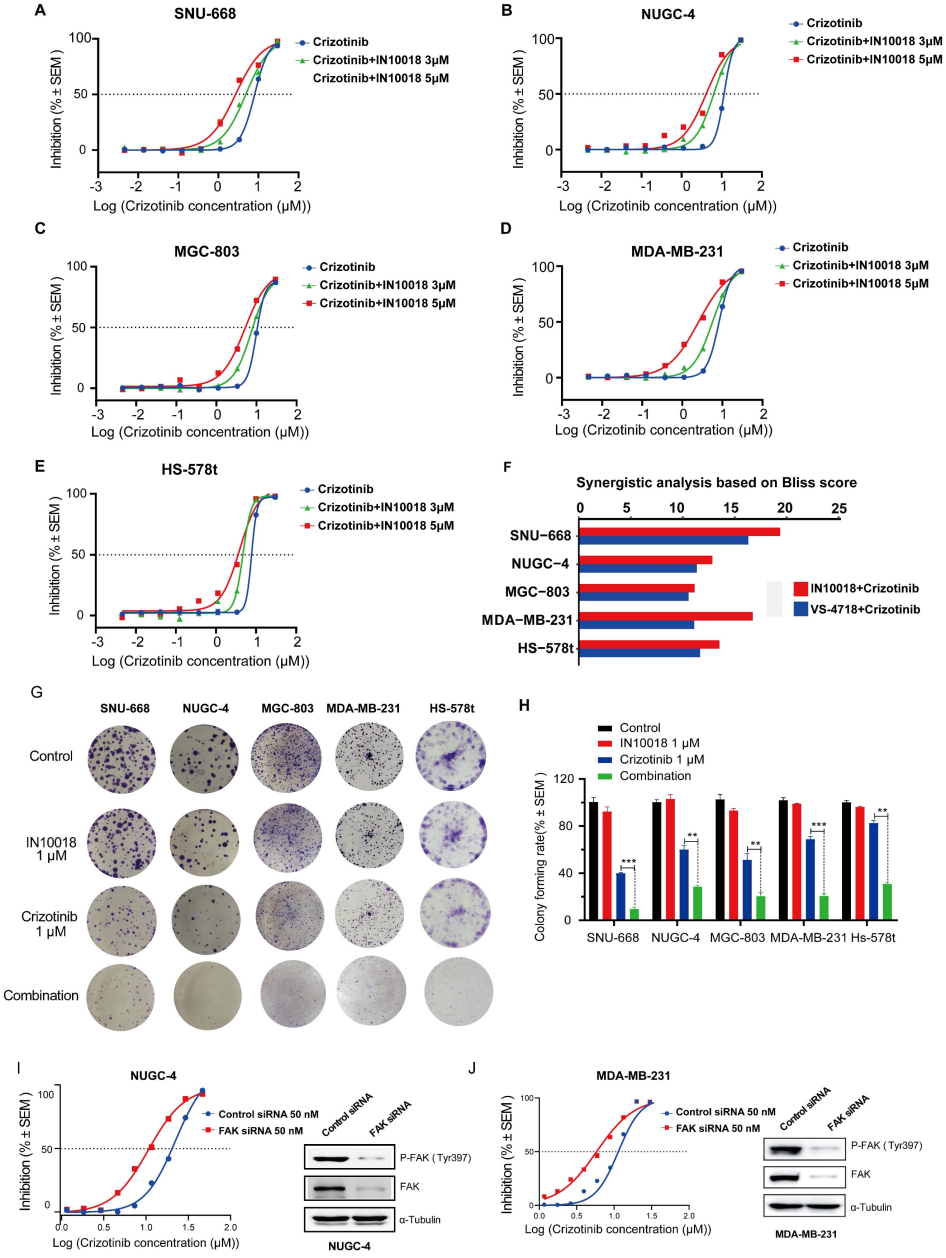
** Co-treatment with FAK inhibition plus crizotinib synergistically inhibits growth of *CDH1*-deficient cancer cells.** (A-E) Cells were treated with various concentrations of crizotinib with or without IN10018 (3 μM or 5 μM) for 72 h. Cell viability was determined by the CellTiter-Glo luminescent assay. Data represent mean ± SEM; n ≥ 3. (F) SynergyFinder 2.0 software was used for Bliss model analysis, with a Bliss score >10 suggesting synergistic effects for the drug combination. (G) Cell clonogenic assay results for co-treatment with crizotinib plus IN10018. Representative images of single-cell clone proliferation, stained with crystal violet. (H) Quantification of the results (G). Data are presented as the mean ± SEM of three independent experiments. *P < 0.05, **P < 0.01, and ***P < 0.001 compared with control cells. (I, J) Knockdown of FAK results in enhanced inhibition of cell viability for NUGC-4 and MDA-MB-231 cells treated with crizotinib. Cells were transfected with control or FAK siRNA, and crizotinib was added 24 h later. Cell viability was evaluated 72 h later. Data represent mean ± SEM; n = 4.

Based on our above findings of p-FAK upregulation under ROS1 inhibitor resistance, we next tested whether inhibiting FAK activity could restore the negative impact of ROS1 inhibitors on cancer cell proliferation *in vitro* using CTG assays in the five *CDH1*-deficient cell lines. We found that monotherapy treatments with the FAK inhibitors IN10018 and VS-4718 resulted in moderate cancer cell killing effects (Figure S2 A-E). Further tests of these FAK inhibitors (3 or 5 μM) combined with crizotinib or entrectinib (30 μM to 4.572 nM) resulted in the dose-dependent inhibition of cancer cell growth in non-resistant *CDH1*-deficient cell lines that was stronger than either monotherapy (Figure 2 A-F, Figure S2 F-U). The corresponding IC50 values for each cell survival curve were summarized in Table S3. These apparently synergistic effects of the combined treatment were evaluated using Bliss scores. To test the long-term (10-day) effects of the crizotinib plus IN10018 drug combination, colony formation assays were also conducted with non-drug-resistant *CDH1*-deficient cell lines. Consistent with the aforementioned results in short-term assays, significantly fewer colonies were consistently observed in the combined treatment group of each cell line compared with that in the monotherapy groups (Figure 2 G-H). Furthermore, FAK knockdown by siRNA in both NUGC-4 and MDA-MB-231 cell lines resulted in higher anti-tumor potency of the crizotinib monotherapy (Figure 2 I and J).

We then examined whether the IN10018 and crizotinib combination also showed enhanced effects in the crizotinib-resistant cell lines. CellTiter-Glo Luminescent Cell Viability (CTG) assays similarly showed significantly greater cell killing following combination treatment in crizotinib-resistant lines compared with that induced by monotherapies, suggesting that the addition of IN10018 restored sensitivity to crizotinib in resistant cells (Figure S3 A-H). Collectively, these data indicated that FAK activation was related to ROS1 inhibitor (crizotinib)-resistance in *CDH1*-deficient cancer cells, and that FAK inhibition could thus overcome this drug resistance, ultimately leading to synergistic therapeutic effects in these cancers.

### FAK inhibitor enhances the anti-tumor effects of ROS1 inhibitor in *CDH1*-deficient cancers *in vivo*

**Figure 3:**
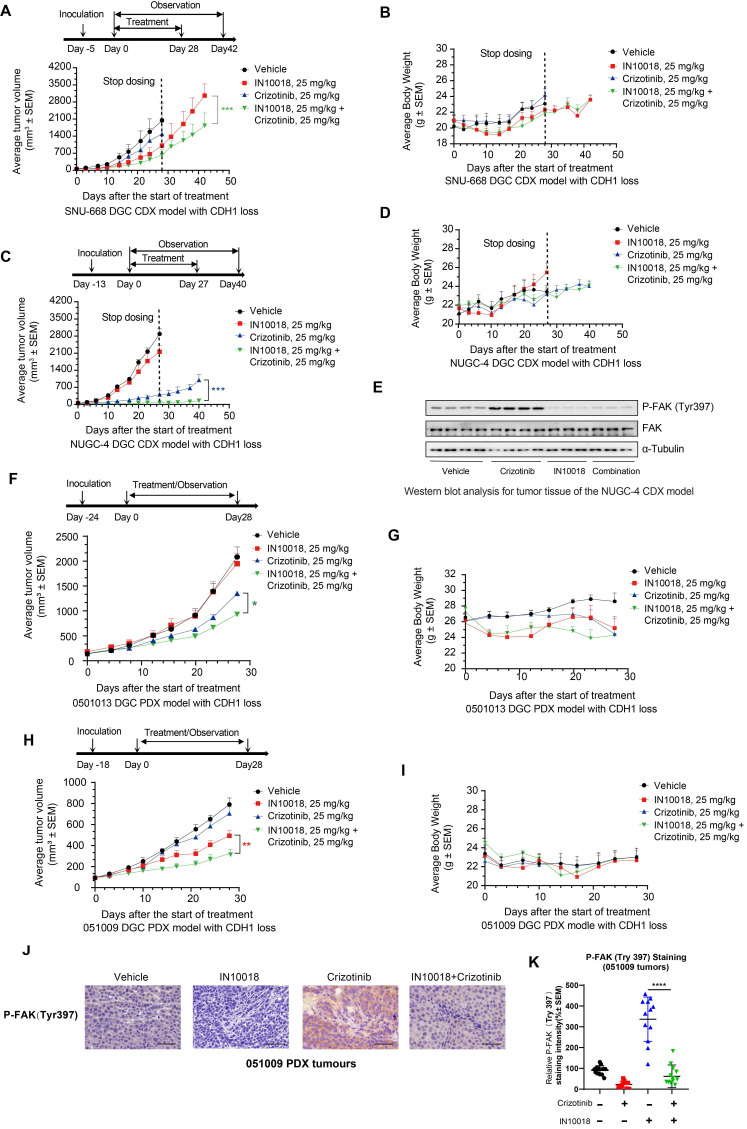
***In vivo* effects of IN10018 plus crizotinib on *CDH1*-deficient cell line-derived xenograft (CDX) and patient-derived xenograft (PDX) models.** (A-D) Tumor growth and mouse body weight changes in mice with SNU-668 and NUGC-4 xenografts treated with the indicated agents. Mice were orally administered vehicle control (0.5% Natrosol 250 HX), 25 mg/kg of IN10018, or 25 mg/kg of crizotinib once daily. Tumor sizes and mouse body weights were recorded twice per week. Data represent mean ± SEM; n ≥ 5. Comparisons were conducted using unpaired student's T-tests. *P < 0.05, **P < 0.01, and ***P < 0.001. Synergy P value is shown. (E) Western blot analysis for FAK signaling in tumor tissue of the NUGC-4 CDX model. (F-I) Tumor growth and mouse body weight changes in the 0501013 and 051009 PDX models. (J, K) P-FAK (Tyr 397) IHC staining for the 051009 tumors. Scale bar = 50 µm. (K) Quantification of the expression levels of P-FAK (Tyr 397) in 051009 tumors from (J). (Data represent Mean ± SEM, n≥12). Statistics analysis was done using unpaired student's T-test. *P < 0.05, **P < 0.01, ***P < 0.001 and ****P < 0.0001.

In light of the above *in vitro* synergistic anti-tumor effects of the combination treatment, we next generated two human *CDH1*-deficient cell line (SNU-668 and NUGC-4)-derived xenograft (CDX) models in mice to evaluate the potential *in vivo* therapeutic effects of the ROS1 inhibitor-IN10018 combination. Administration of either crizotinib, entrectinib, or IN10018 monotherapies (25 mg/kg, p.o., daily) resulted in relatively limited tumor inhibition compared with that following administration of the drug combinations, which led to significantly attenuated tumor development (Figure 3 A-D). Furthermore, inhibition of tumor growth was sustained for 2 weeks beyond the end of the dosing period under the combination regimen (Figure 2 A, C), and entrectinib showed comparable anti-tumor effects in the NUGC-4 and SNU-668 CDX models (Figure S4 A-D). All treatments were well tolerated in this study (Figure 3 A-D and Figure S4 A-D). After completion of the treatment period, Western blot analysis of tumor samples harvested from NUGC-4 CDX model mice showed that p-FAK was upregulated to significantly higher levels in the crizotinib monotherapy group compared with that in the combination treatment group (Figure 3 E), suggesting that FAK inhibition by IN10018 administration could suppress the activation of FAK signaling associated with crizotinib.

We then evaluated the *in vivo* effects of the co-administration of crizotinib with a FAK inhibitor in two *CDH1*-deficient DGC patient-derived xenograft (PDX) models, 0501013 and 051009. Similar to the results obtained in CDX model mice, tumor volumes in the group treated with crizotinib (25 mg kg^-1^, p.o.) plus IN10018 (25 mg kg^-1^, p.o., daily) for 4 weeks were significantly smaller than those in either the monotherapy or vehicle control groups (Figure 3F-I). Mice displayed good tolerance for all treatments during the drug dosing period (Figure 3 F-I). IHC analysis of tumor samples collected from the 051009 PDX model at the end of therapy showed that P-FAK expression levels were significantly higher in the crizotinib monotherapy compared to the combination therapy, which was consistent with the results obtained in the NUGC-4 tumors (Figure 3 J, K). These cumulative findings indicated that targeting ROS1 with either crizotinib or entrectinib, in conjunction with IN10018-mediated FAK inhibition, enhanced the *in vivo* anti-tumor effects of either monotherapy in both CDX and PDX *CDH1*-deficient mouse models.

### Inhibiting FAK enhances the oxidative stress-induced DNA damage caused by crizotinib via regulation of the YAP-TRX axis

**Figure 4:**
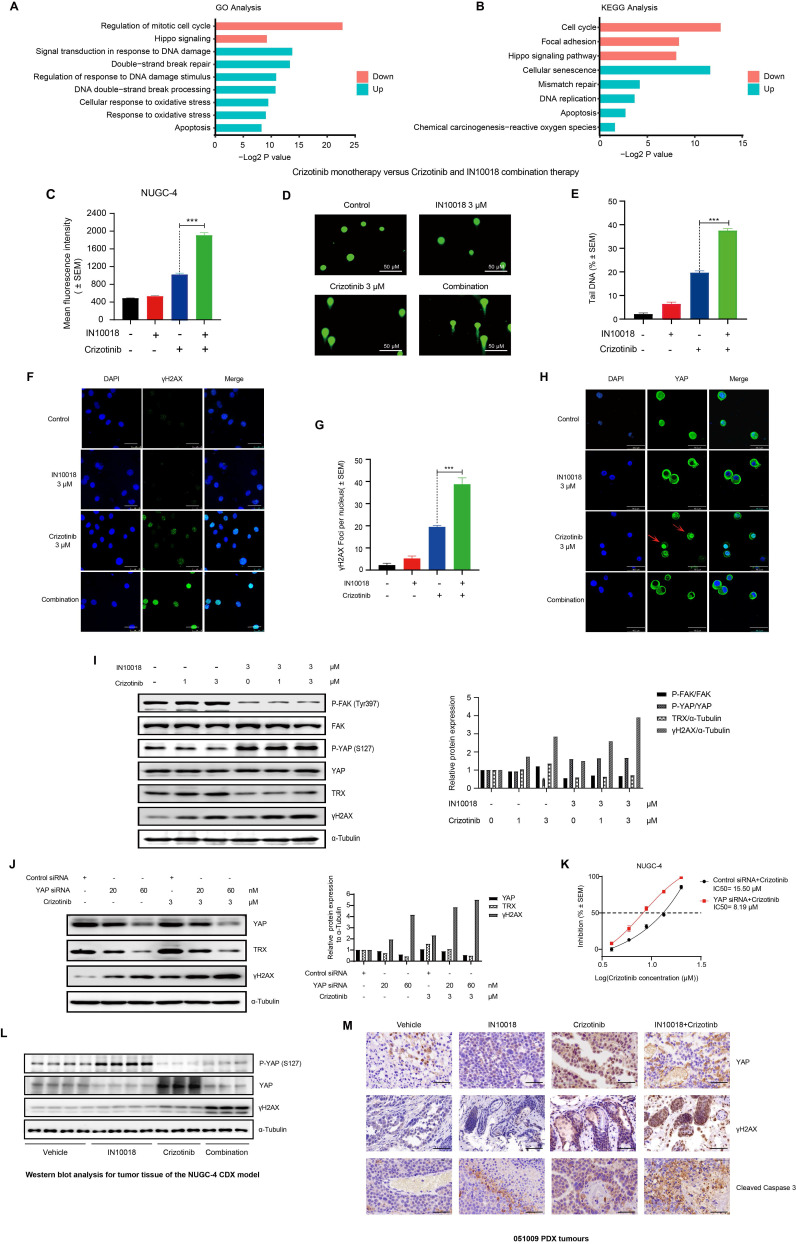
** Reactive oxygen species (ROS) accumulation and DNA damage is enhanced by addition of IN10018 with crizotinib vs crizotinib alone.** (A, B) GO and KEGG enrichment analysis of significantly up-regulated and down-regulated genes between crizotinib monotherapy group and combination treatment group. The Y-axis represents the name of the pathway, and the X-axis represents the P value with the log2-transformed in enriched pathways, the different colors of bar represent pathways that were significantly enriched for significantly up-regulated and down-regulated genes in two groups. (C) Intracellular ROS production as assessed by DCFH-DA fluorescence flow cytometry. Quantitative histograms of the mean fluorescence intensity (M.F.I.) values (D) of n ≥ 5 independent FACS experiments are shown (Data represent mean ± SEM). The NUGC-4 cells were treated with 3 μM crizotinib with or without 3 μM IN10018 (as indicated) for 6 h. (D, E) DNA damage in NUGC-4 cells as assessed by the comet assay following treatment with 3 μM crizotinib with or without 3 μM IN10018 (as indicated) for 6 h. (E) Quantification of the results (D) (Data are presented as the mean ± SEM, n=100. *P < 0.05, **P < 0.01, and ***P < 0.001 compared with crizotinib-treated cells). (F, G) DNA damage as detected by γH2AX immunofluorescence following treatment with 3 μM crizotinib with or without 3 μM IN10018 (as indicated) for 6 h. Scale bars = 25 μm. (G) Quantification of the results (F), n=100. Data are presented as the mean ± SEM of the number of γH2AX foci per nucleus from three separate experiments. *P < 0.05, **P < 0.01, and ***P < 0.001 compared with crizotinib-treated cells. (H) YAP immunofluorescence image following treatment with 3 μM crizotinib with or without 3 μM IN10018 (as indicated) for 48 h. Scale bars = 46.2 μm. (I) Western blot analysis of YAP/TRX signaling and DNA damage markers following treatment with 3 μM crizotinib with or without 3 μM IN10018 (as indicated) for 6 h. (J) Western blot analysis of YAP immunofluorescence and DNA damage following treatment with 3 μM crizotinib for 6 h. (K) Inhibition of cell growth in NUGC-4 cells with YAP knockdown treated with crizotinib. (L) Western blot analysis for YAP signaling in tumor tissue of the NUGC-4 CDX model. (M) YAP, γH2AX and Cleaved Caspase 3 IHC staining for the 051009 tumors. Scale bar = 50 µm.

To explore which pathways might be responsible for the synergistic effects of FAK/ROS1 inhibition, we conducted whole transcriptome profiling (RNA-seq) in NUGC-4 cells treated for 24 h with crizotinib, IN10018, the crizotinib-IN10018 combination, or vehicle control. Gene Ontology and KEGG analysis revealed that genes differentially expressed between crizotinib monotherapy and the combination therapy were primarily enriched in pathways related to DNA damage, oxidative stress, and Hippo signaling (Figure 4 A, B and Figure S5 A, B). To verify the involvement of these processes, we performed diacetyldichlorofluorescein diacetate (DCF) fluorescence flow cytometry, comet assays, and γH2AX expression assays to evaluate ROS accumulation and DNA damage. The results of these assays showed that oxidative stress and DNA damage were greater in the combined treatment group compared with the crizotinib monotherapy group (Figure 4 C-G and Figure S5 C).

YAP is an essential transcriptional co-factor for Hippo pathway signaling, and aberrant regulation of YAP activity has been linked to the occurrence of drug resistance in cancer treatments 29. FAK controls YAP signaling by regulating its translocation to the nucleus and subsequent activation 30. Further examination of our RNA-seq data revealed that FAK-mediated YAP signaling could potentially participate in the mechanism responsible for crizotinib resistance. We also used RT-qPCR and western blot analysis to test the expression of YAP signaling downstream genes in NUGC-4 cells. Crizotinib monotherapy induced activation of the YAP signaling pathway, which can be significantly suppressed by combination therapy (Figure S5 D, E). Notably, the nuclear localization of YAP was significantly increased at 48 h of exposure to crizotinib, suggesting that the regulation of YAP signaling may be abnormal (Figure 4 H and Figure S5 F). Treatment with the FAK inhibitor IN10018 combined with crizotinib resulted in obvious YAP export from the nucleus (Figure 4 H and Figure S5 F). Detection of the ROS scavenger TRX, a downstream biomarker of YAP 31, showed its concomitant downregulation in the group treated with IN10018 (Figure 4 I), which could explain the heightened accumulation of ROS in the combined treatment group compared with that in the crizotinib monotherapy group. Crizotinib has been shown to trigger ROS accumulation, which can in turn directly induce DNA damage 32. Consistent with this effect, we observed that exposure to a ROS1 inhibitor induced γH2AX expression, which was further increased in cells treated with the combination therapy in a crizotinib dose-dependent manner (Figure 4 I). We then assessed whether the increased DNA damage was dependent on YAP-TRX expression in cells treated with crizotinib. Knockdown of YAP by siRNA also led to reduced TRX expression, further supporting the role of YAP-regulated TRX expression. Reduced TRX expression coincided with elevated γH2AX levels under YAP knockdown (Figure 4 J). Moreover, cell killing and DNA damage were both markedly increased under crizotinib treatment in NUGC-4 cells with YAP knocked down (Figure 4 K and Figure S5 G, H).

To further validate the role of the mechanistic results *in vitro*, we evaluated the YAP signalling and DNA damage *in vivo*. IHC staining and western blot analysis of tumor samples from NUGC-4 showed that crizotinib group tumours exhibited high level expression of YAP in nuclear, which was significantly suppressed in the combination therapy tumours. (Figure 4 L and Figure S6 A,B).

We also note that more DNA damage and apoptosis was induced by the combination therapy than by the crizotinib monotherapy (Figure 4 L and Figure S5 A,B). The IHC staining of the 051009 tumors showed comparable results of the NUGC-4 tumors (Figure 4 M and Figure S6 C). Taken together, these results suggested that FAK inhibition led to increased DNA damage induced by crizotinib through regulation of the FAK-YAP axis* in vitro* and* in vivo*.

### Exogenous N-acetylcysteine (NAC) or YAP activation mitigates the anti-tumor effects of FAK/ROS1 inhibition

**Figure 5:**
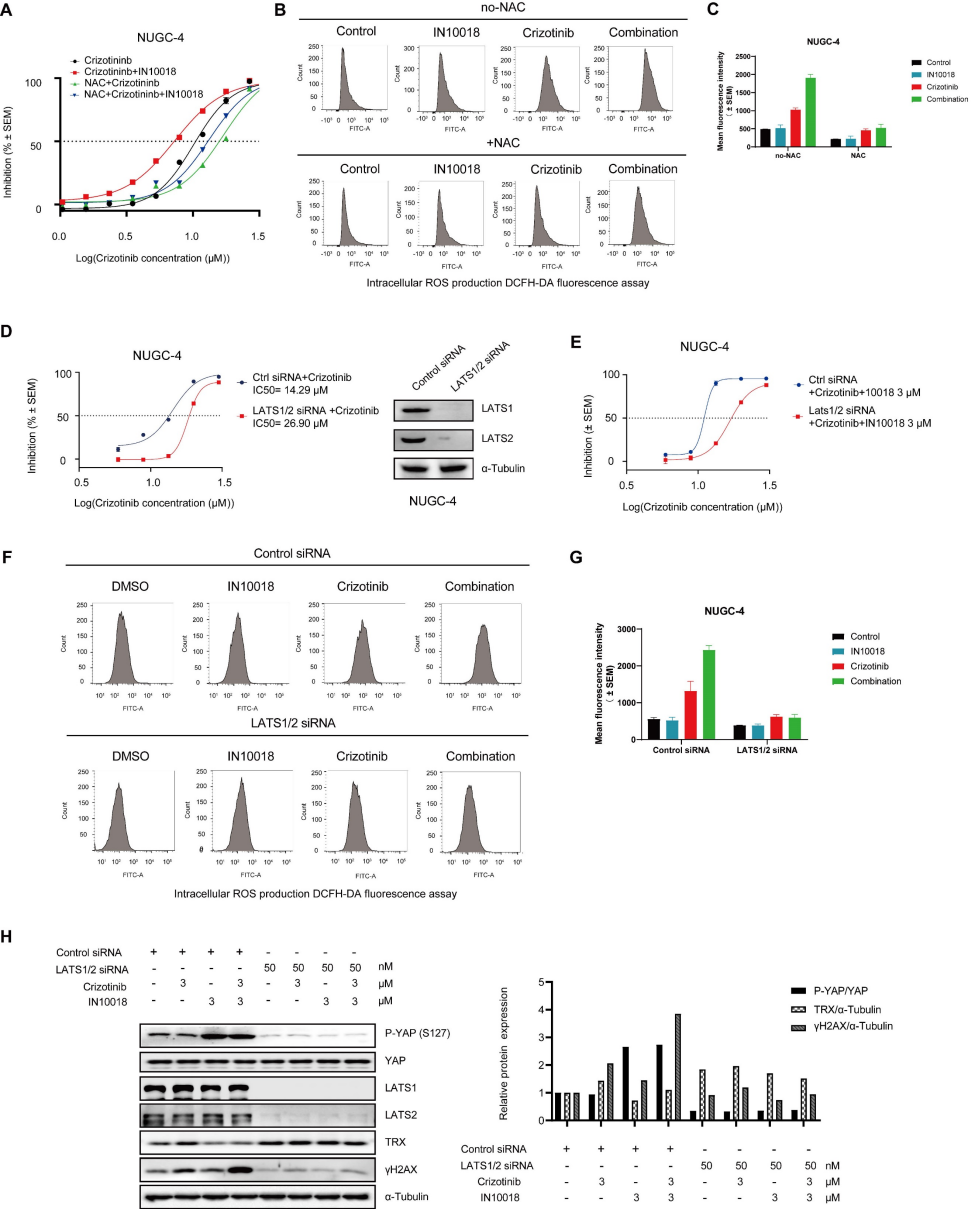
** Antioxidant N-acetylcysteine (NAC) and activation of YAP rescue cell killing induced by co-treatment of crizotinib and IN10018.** (A) NUGC-4 cells were pretreated with 5 mM NAC for 24 h before the addition of crizotinib, IN10018, or both for 48 h. (B, C) Intracellular production of reactive oxygen species (ROS) assessed by DCFH-DA fluorescence cytometry. NUGC-4 cells were pretreated with NAC for 24 h. After removal of NAC, cells were treated with 3 μM crizotinib or 3 μM IN10018 or both (as indicated) for 12 h. (C) Quantification of the results(B) (Data represent mean ± SEM of three independent experiments; n = 4). (D, E) Knockdown of LATS1/2 in NUGC-4 cells enhances cell resistance to treatment with crizotinib alone or with IN10018. Cells were transfected with control siRNA or LATS1/2 siRNA. After 48 h, crizotinib alone or plus IN10018 was added. Cell viability was evaluated 72 h later (Data represent mean ± SEM; n = 4). (F, G) Intracellular production of ROS assessed by DCFH-DA fluorescence cytometry. LATS1/2 was knocked down in NUGC-4 cells, which were then treated with 3 μM crizotinib, 3 μM IN10018, or both (as indicated) for 12 h. (G) Quantification of the results (F) (Data represent mean ± SEM of three independent experiments, n=4). (H) Knockdown of LATS1/2 in NUGC-4 cells. Western blot analysis for YAP/TRX signaling and DNA damage after treatment with 3 μM crizotinib for 6 h.

To confirm whether the cell killing effects of the combination therapy were dependent on oxidative stress, we assessed whether exogenous application of the ROS scavenger NAC protected NUGC-4 cells from the oxidative damage induced by the co-treatment of IN10018 and crizotinib in vitro. Cells pre-treated for 12 h with NAC (5 mM) and then exposed to the crizotinib-IN10018 combination therapy showed significantly lower cell death than those in the combination treatment group without pretreatment, further supporting a role of oxidative stress in the cell killing effects of the combination therapy (Figure 5 A-C).

Because LATS1/2 phosphorylates YAP in the cytoplasm to block its localization to the nucleus and consequently downregulate its activity, we hypothesized that suppressing LATS1/2 expression would activate YAP signaling in the nucleus by limiting its retention in the cytoplasm. To test this hypothesis, we generated siRNA-induced LATS1/2 knockdown in the NUGC-4 cell line (Figure 5 D). The resulting cells displayed significantly less cell death and ROS accumulation in response to the combination therapy than the scrambled siRNA control cells under the same conditions (Figure 5 D-G). Knockdown of LATS1/2 by siRNA also led to upregulated TRX expression and induced a lower level of γH2AX, which coincided with the inhibition of cell death (Figure 5 H). These results further supported the likelihood that suppression of oxidative stress response by inhibiting the FAK/YAP/TRX pathway and ROS1 inhibition was indeed responsible for the anti-tumor effects of the combination treatment.

### ROS1-dependent effects of FAK signaling activation

**Figure 6:**
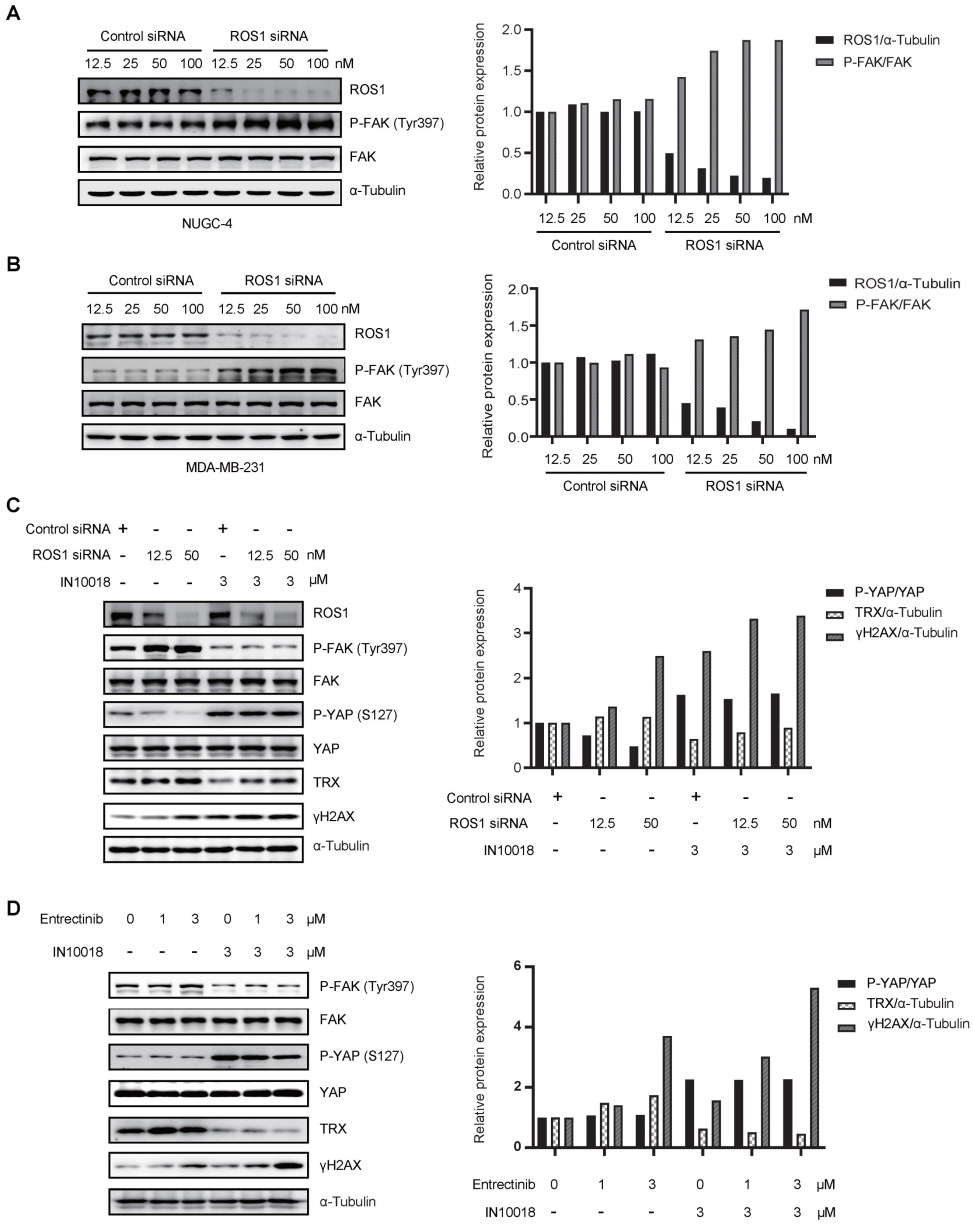
** Activation of FAK signaling and enhanced DNA damage depend on ROS1, not on anaplastic lymphoma kinase (ALK).** (A, B) Western blot analysis for FAK signaling and downstream markers of ROS1 after siRNA treatment of NUGC-4 and MDA-MB-231 cells. (C) Western blot analysis for YAP signaling and DNA damage in NUGC-4 cells after treatment with ROS1 siRNA. (D) Western blot analyzes for YAP/TRX signaling and DNA damage following treatment with 3 μM entrectinib, 3 μM IN10018, or both (as indicated) for 6 h.

Crizotinib and entrectinib are non-selective tyrosine kinase inhibitors, and both have been shown to target ALK and ROS1. To exclude the potential influence of off-target effects, we generated siRNA-induced ROS1 and ALK knockdown cell lines on the NUGC-4 genetic background. Western blot analysis with band density quantification showed that ROS1 knockdown, but not ALK knockdown, induced hyperactivation of FAK (Figure 6 A-B and Figure S7 A-B). Moreover, cells with ROS1 knockdown treated with IN10018 exhibited higher levels of γH2AX than cells with ROS1 knockdown without FAK inhibitor treatment, which was consistent with the results obtained following ROS1 inhibitor treatment (Figure 6 C and Figure S7 C). In addition, we observed that DNA damage was substantially increased in cells treated with entrectinib plus a FAK inhibitor (Figure 6 D). These results thus demonstrated that crizotinib- or entrectinib-induced activation of FAK signaling and the subsequent enhanced DNA damage depend on ROS1, but not on ALK, inhibition in CDH1-deficient cells.

## Discussion

DGC and TNBC remain aggressive diseases without effective targeted therapies 5, 6. Both of these cancers feature *CDH1* gene deficiency, and synthetic lethality between ROS1 and *CDH1* has already been established, providing a potential clinical treatment strategy for patients with these types of cancer 7. On the basis of preclinical findings, two clinical trials, the ROLO study (NCT03620643) and the ROSALINE study (NCT04551495), are being conducted to assess the efficacy of co-administering a ROS1 inhibitor with either crizotinib or entrectinib in advanced DGC and TNBC featured with *CDH1* deficiency 10, 11. However marked and durable the initial response may be to treatment with crizotinib, the vast majority of patients with non-small cell lung cancer will develop resistance within a few years 12. Therefore, it is necessary to explore a therapeutic regimen to overcome ROS1 inhibitor-resistance in DGC and TNBC.

As a key coordinator of cellular responses to environmental stresses, FAK is an attractive target supporting tumorigenesis processes and resistance mechanisms. The inducible product of FAK tyrosine phosphorylation is enhanced by therapeutic stress to resist the deleterious effects of tumor therapy, including MEK inhibitors and KRAS-G12C inhibitors 23, 24. In the present study, hyperactivation of FAK signaling was also found to be related to the adaptive resistance to a ROS1 inhibitor in the treatment of *CDH1*-deficient cancers. In addition, administration of a FAK inhibitor promoted the anti-tumor effect of ROS1 inhibitors for both original and crizotinib-resistant *CDH1*-deficient cancer cells.

Rational combination therapy may enhance therapeutic efficacy by overcoming primary and acquired resistance. Combination therapy that includes a FAK inhibitor has shown synthetic anti-tumor effects and has overcome drug resistance in preclinical studies 33, 34. IN10018 (formerly known as BI 853520) is a specific FAK kinase inhibitor that is currently under evaluation in a number of phase 1/2 clinical trials 35, 36. Considering the synergistic effects of the drug combinations tested in the present study in vitro, we assessed the combination therapy of IN10018 with ROS1 inhibitors in vivo. Co-treatment with IN10018 and ROS1 inhibitors significantly reduced the tumor size in CDX models and PDX models with *CDH1* deficiency. We also observed that monotherapy with crizotinib induced FAK activation in the NUGC-4 CDX model. These results suggested a potential clinical benefit from the co-administration of a FAK inhibitor with the clinically available crizotinib or entrectinib for patients with *CDH1*-deficient cancer.

Previous reports have indicated that crizotinib reduces cell growth through the accumulation of intracellular oxidative stress and DNA damage in gastric cancer and in human liver cells 32, 37. Adaptively enhanced anti-oxidative signaling is one mechanism whereby anti-tumor effects may be compromised. TRX has been suggested for use in chemotherapy to overcome oncogene-induced senescence by preventing ROS accumulation 38, 39. Transcriptome profiling results have suggested that IN10018 in combination with crizotinib induces stronger oxidative stress-related DNA damage and affects the activity of the Hippo pathway. The FAK-YAP signaling axis is a well-known pathway in the development of cancer 40. YAP is located upstream of antioxidant enzymes, especially for TRX 31, 41. We thus considered whether FAK-YAP signaling further enhanced TRX activity to decrease the effect of the ROS1 inhibitor. Indeed, ROS1 inhibition by either crizotinib or entrectinib increased FAK-YAP activity and enhanced TRX activity, which decreased oxidative stress-related DNA damage to compromise the anti-tumor effect of the drug treatment. This mechanism was confirmed by our rescue studies with the antioxidant NAC and by transfection studies with knockdown of LATS1/2, which acted as negative regulator of YAP signaling. Taken together, our findings indicated that FAK inhibition enhanced the effects of ROS1 inhibitors through regulation of the FAK-YAP-TRX signaling axis.

Crizotinib and entrectinib, as multi-target tyrosine kinase inhibitors, similarly induce FAK activation and have two identical targets: ROS1 and ALK. To determine whether ALK, ROS1, or both contributed to FAK activation, ALK and ROS1 were knocked down using their respective siRNAs. We found that knockdown of ROS1, but not of ALK, specifically enhanced the phosphorylation of FAK. Furthermore, the combination of ROS1 siRNA with IN10018 effectively attenuated YAP-TRX signaling, promoting oxidative stress-related DNA damage.

In conclusion, our results indicate that ROS1 inhibition through the administration of either crizotinib or entrectinib compromised cancer treatment outcomes by blunting oxidative stress-related DNA damage through the stimulation of the FAK-YAP-TRX signaling pathway. Co-administration of the FAK inhibitor IN10018 enhances the anti-tumor effect of ROS1 inhibitors in the treatment of *CDH1*-deficient cancers.

## Supplementary Material

Supplementary figures and tables.

## Abbreviations

DGC: Diffuse gastric cancer
TNBC: Triple-negative breast cancer
FAK: Focal adhesion kinase
ROS1: ROS proto-oncogene 1 receptor tyrosine kinase
TRX: Thioredoxin
ROS: Reactive oxygen species
NAC: N-acetylcysteine
