# Supplementary material for: Synergism of FAK and ROS1 inhibitors in the treatment of CDH1-deficient cancers mediated by FAK-YAP signaling
Source: Int J Biol Sci. 2023 May 15;19(9):2711–24. doi: 10.7150/ijbs.81918 (PMC10266074; doi:10.7150/ijbs.81918)
Supplement: Supplementary file 1 — Supplementary figures and tables. [file ijbsv19p2711s1.pdf]

Supplementary materials

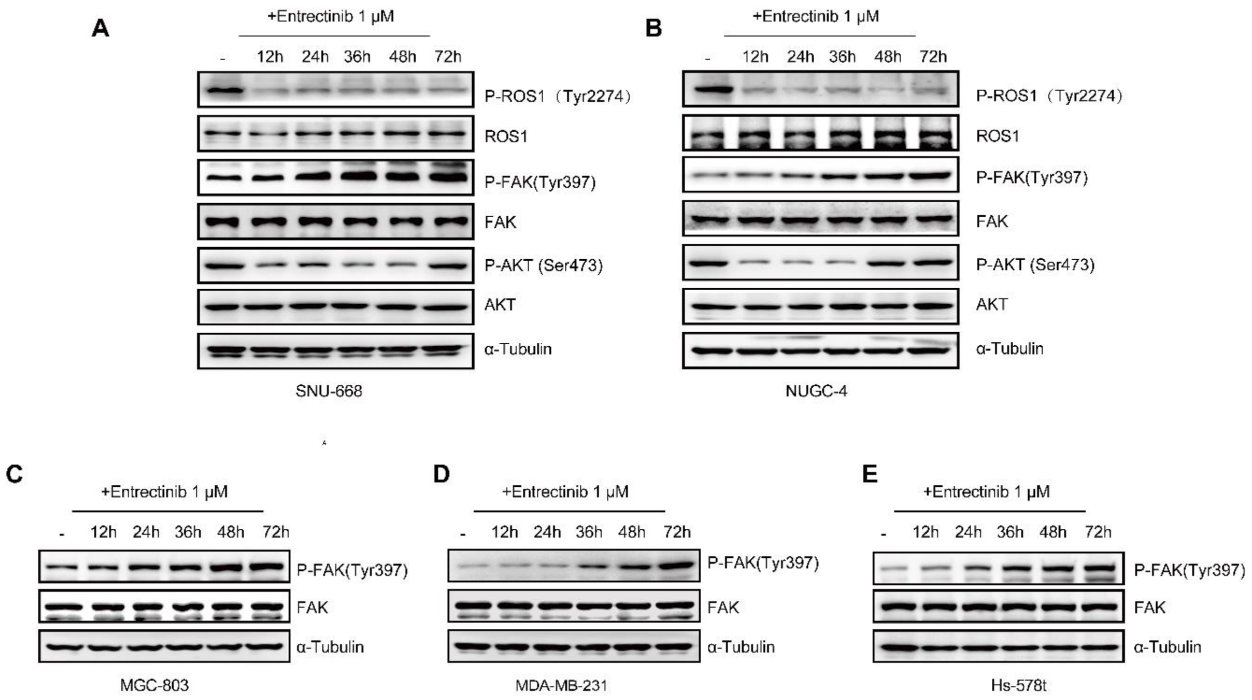

Figure S1. FAK signaling activation is adaptively induced by entrectinib

(A-E) Western blot analysis for FAK signaling and downstream markers after entrectinib treatment for various times. Total protein was extracted for Western blotting.

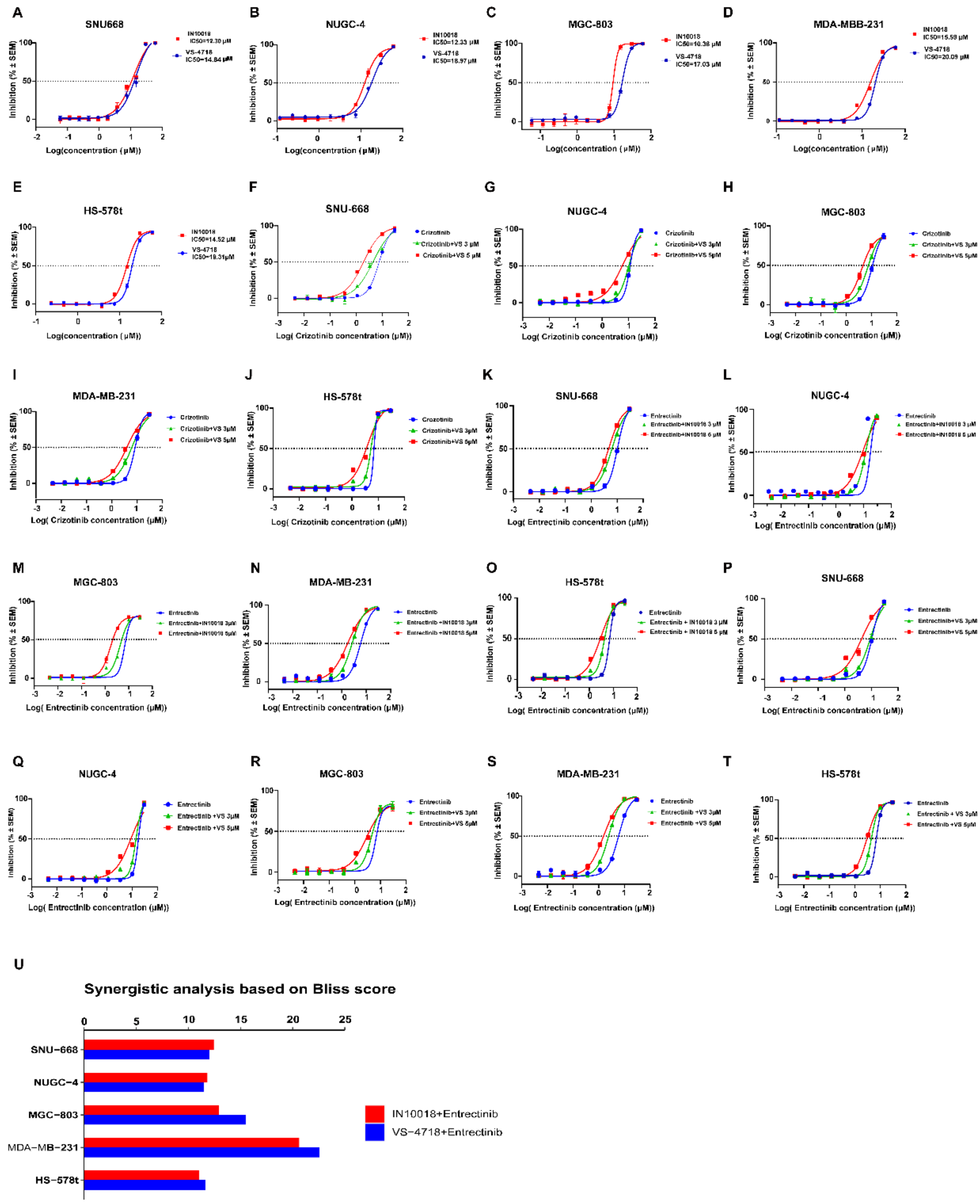

Figure S2. Co-treatment with crizotinib or entrectinib plus FAK inhibitors inhibits growth of *CDH1*-deficient cancer cells better than either monotherapy

(A-E) Inhibition of cell growth was assessed following FAK inhibitor monotherapy. Cells were treated with various concentrations of IN10018 and VS-4718 for 72 h. (F-T) Assessment of cell viability after co-treatment with ROS1 inhibitors and a FAK inhibitor. Non-resistant *CDH1*-deficient cells were incubated with various concentrations of ROS1 inhibitors with or without a FAK inhibitor (3 μM or 5 μM) for 72 h. Cell viability was determined using the CellTiter-Glo luminescent assay. The IC50 value was shown in Table

S3. Data represent mean ± SEM, n ≥ 3. (F-J) Analysis of co-treatment with crizotinib and VS-4718. (K-T) Analysis of co-treatment with entrectinib and IN10018 or VS-4718. (U) Bliss analysis using SynergyFinder 2.0 software, with a Bliss score >10 suggesting synergistic effects for the drug combination.

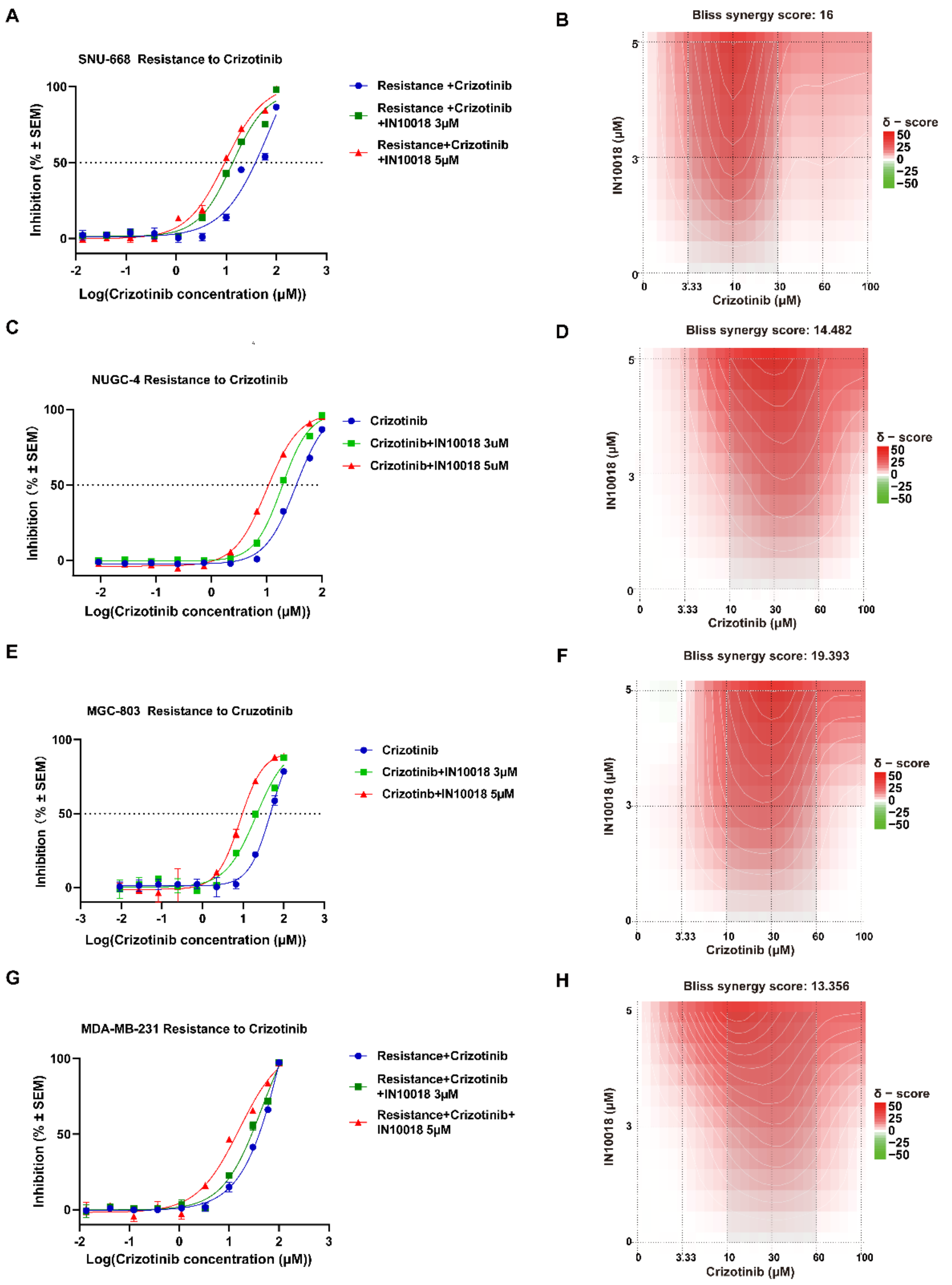

Figure S3. Crizotinib and IN10018 co-therapy has synergistic effects in crizotinib-resistant cell lines.

(A-H) Effects of co-treatment with IN10018 and crizotinib on crizotinib-resistant cell lines. Cells were co-treated with IN10018 (3 μM or 5 μM) and a serial dilution of 100 μM crizotinib for 72 h. Cell viability was determined using the CellTiter-Glo luminescent assay. Data represent mean ± SEM; n ≥ 3. (B, D, F, H) Bliss analysis was conducted with SynergyFinder,

with a Bliss score >10 suggesting synergistic effects for the drug combination. The Bliss synergy scores represent overall synergies for combination.

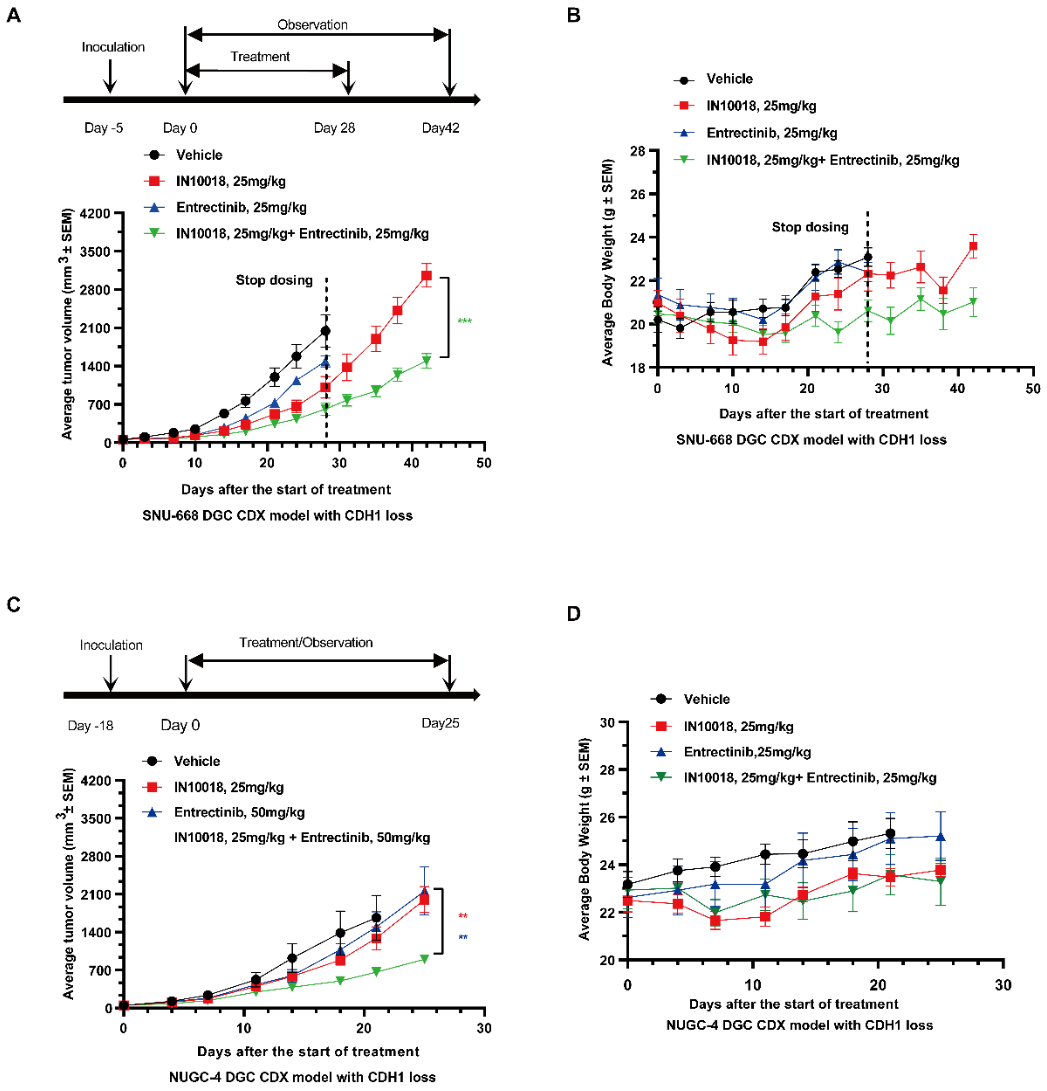

Figure S4. *In vivo* evaluation of the effects of IN10018 and entrectinib co-treatment on *CDH1*-deficient CDX models.

(A-D) Vehicle control (0.5% Natrosol 250 HX) or 25 mg/kg of IN10018 and 25 mg/kg of entrectinib was orally administered to mice once daily. Tumor size and mouse body weight were recorded twice each week. Data represent mean ± SEM; n ≥ 5. Comparisons were performed using unpaired student's T-tests. *P < 0.05, **P < 0.01, and ***P < 0.001.

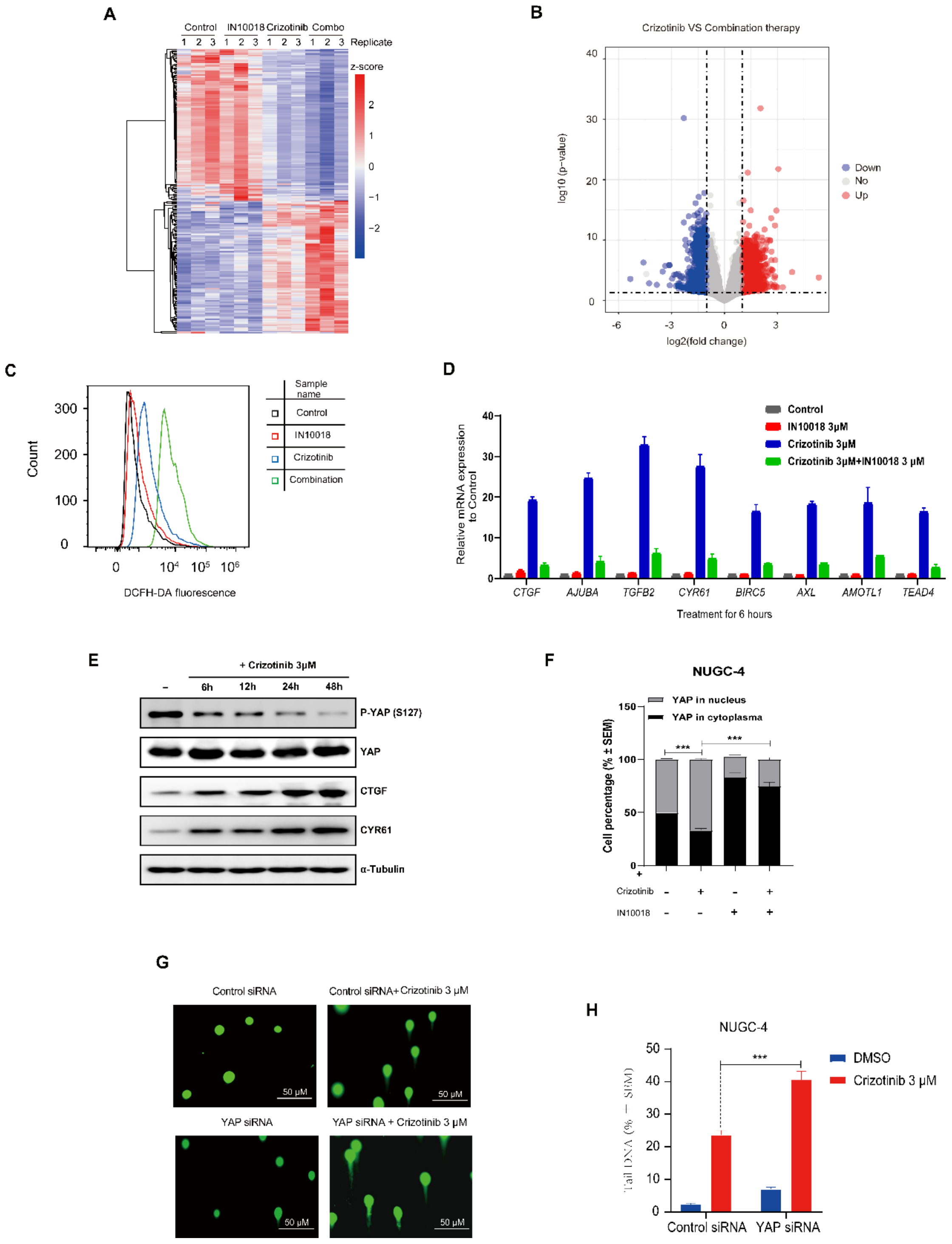

Figure S5. YAP nuclear translocation is suppressed by co-treatment with IN10018 plus crizotinib *in vitro*.

(A) Heatmap of genes differentially expressed after crizotinib monotherapy and after co-treatment with crizotinib plus IN10018. NUGC-4 cells were incubated with 1 μM crizotinib, 1 μM IN10018, or both (as indicated) for 24 h. (B) Volcano plot showing 2487 differentially expressed genes. Red (n = 1466) and blue (n = 1021) represent genes up-regulated and

down-regulated, respectively, in the co-treated group relative to the crizotinib-treated group. (C) Intracellular ROS production as assessed by DCFH-DA fluorescence flow cytometry. Representative flow cytometry profiles are shown. NUGC-4 cells were treated with 3 μM crizotinib with or without 3 μM IN10018 (as indicated) for 6 h. (D) Expression levels of YAP signaling signature genes by RT-qPCR. The gene expression data of 6 h treated in NUGC-4 cells which were normalized to the DMSO control group. (Data represent Mean ± SEM, n ≥ 3). (E) Western blot tests on NUGC-4 cells with Crizotinib treatment for 6h. (F) ImageJ analysis of YAP immunofluorescence from Fig 4. Data represent mean ± SEM; n = 50. Comparisons were conducted using unpaired student's T-tests. *P < 0.05, **P < 0.01, ***P < 0.001. (G, H) DNA damage with YAP knockdown in NUGC-4 cells as assessed by the comet assay following treatment with 3 μM crizotinib for 6 h. (H) Quantification of the results (G). Data are presented as the mean ± SEM; n = 100. *P < 0.05, **P < 0.01, and ***P < 0.001 compared with crizotinib-treated cells.

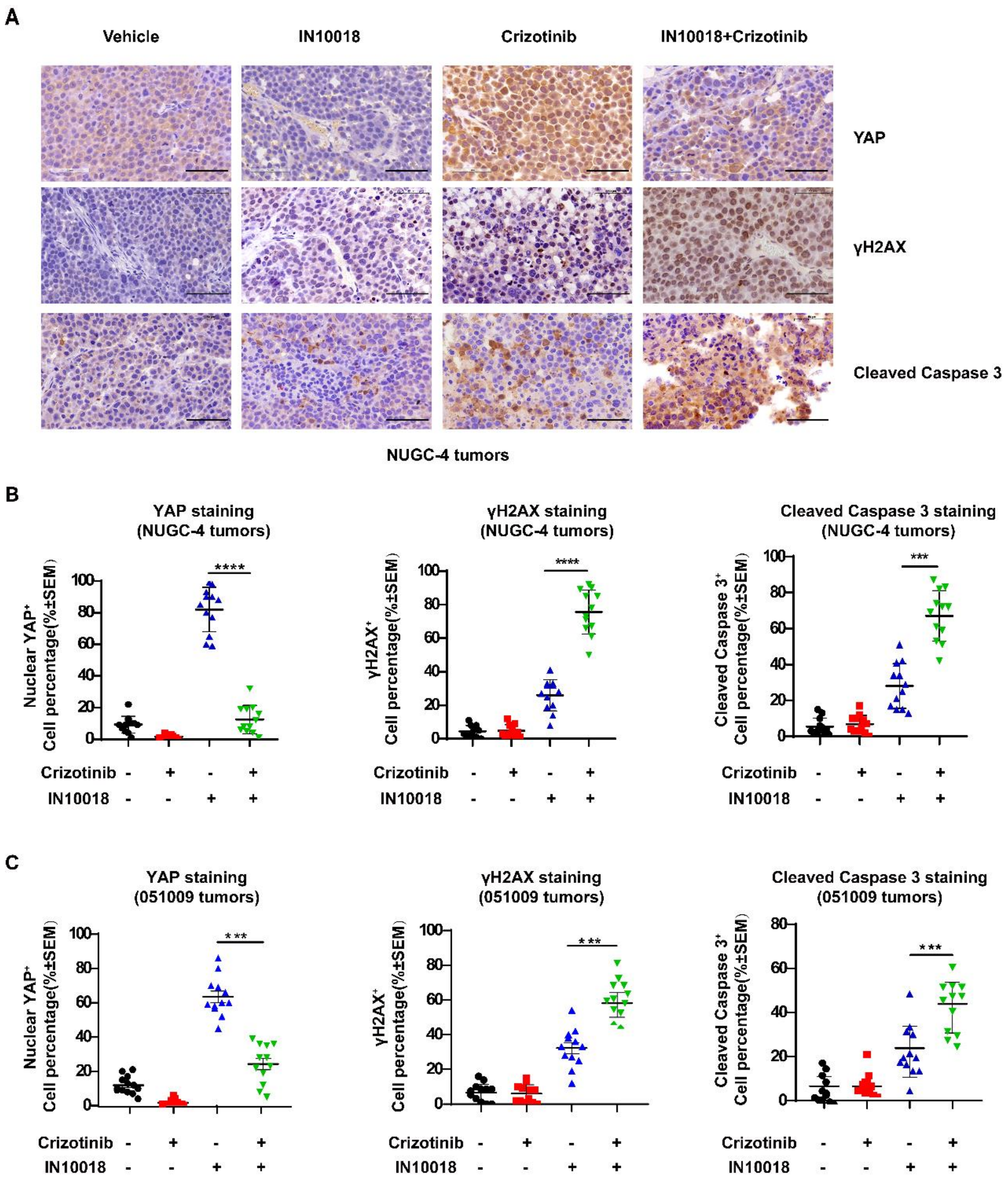

Figure S6. YAP nuclear translocation is suppressed by co-treatment with IN10018 plus crizotinib *in vivo*.

(A, B) YAP, γH2AX and Cleaved Caspase3 IHC staining for the NUGC-4 CDX model tumors. Scale bar = 50 μm. (B) Quantification of the expression levels of YAP, γH2AX and cleaved Caspase3 in NUGC-4 tumors from (A) using ImageJ. (Data represent Mean ± SEM, n≥100). (C) Quantification of the expression levels of YAP, γH2AX and cleaved Caspase3 in NUGC-4 tumors from Figure 4 (M). (Data represent Mean ± SEM, n≥100). Statistical

analysis was done using unpaired student's T-test. *P < 0.05, **P < 0.01, ***P < 0.001 and ****P < 0.0001.

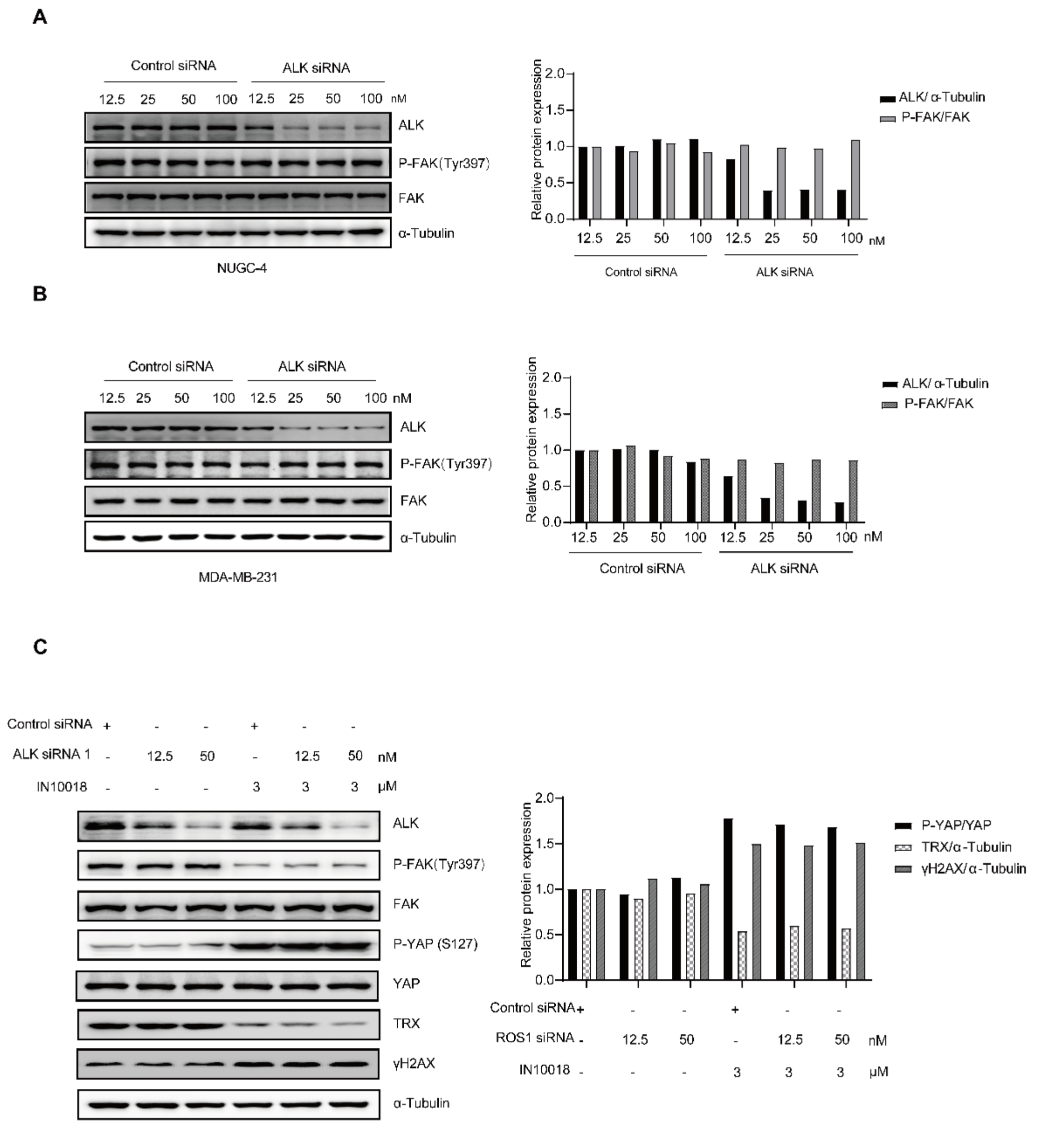

Figure S7. ALK knockdown has little effect on FAK signaling or DNA damage

(A, B) Western blot analysis of FAK signaling and downstream markers following addition of siRNA ALK to NUGC-4 and MDA-MB-231 cells. (C) Western blot analysis of YAP signaling and DNA damage following addition of siRNA ALK to NUGC-4 cells.

Table S1: List of antibodies used in this study.

Antibody	Vendor	Catalogue Number
FAK	Cell Signaling Technology	71433
Phospho-FAK (Tyr397)	Thermo	PA5-17084
YAP	Proteintech	13584-1-AP
Phospho-YAP (Ser127)	Cell Signaling Technology	13008
ROS1	Thermo	PA1-30318
Phospho-ROS1 （Tyr2274）	Cell Signaling Technology	3078
ALK	Cell Signaling Technology	3633
AKT	Cell Signaling Technology	4691
Phospho-AKT (Ser473)	Cell Signaling Technology	4060
TRX	Abcam	ab26320
γH2AX	Cell Signaling Technology	80312
LATS1	Cell Signaling Technology	3477
LATS2	Cell Signaling Technology	5888
α-Tubulin	Proteintech	HRP-66031
Anti-rabbit HRP	Cell Signaling Technology	7074
Goat anti-Rabbit Alexa Fluor™ 488	Thermo	A-11008
DAPI	Thermo	D21490
CYR61	Proteintech	26689-1-AP
CTGF	Proteintech	25474-1-AP
Cleaved caspase 3	Cell Signaling Technology	9661

Table S2: List of siRNA and primers used in this study.

Gene Name	Forward primer (5'-3')	Reverse primer (5'-3')
Control siRNA	UUCUCCGAACGUGUCACGUTT	ACGUGACACGUUCGGAGAATT
FAK siRNA	CCUGUAUGCCUAUCAGCUUTT	AAGCUGAUAGGCAUACAGGTT
Lats1 siRNA	GGUAGUUCGUCUAUAUUAUTT	AUAAUAUAGACGAACUACCTT
Lats2 siRNA	CUACCAGAAAGAGUCUAAUTT	AUUAGACUCUUUCUGGUAGTT
YAP siRNA	GACAUCUUCUGGUCAGAGATT	GGUGAUACUAUCAACCAAATT
CTGF	GTTTGGCCCAGACCCAACTA	GGCTCTGCTTCTCTAGCCTG
AJUBA	AGCCACCAGGTCCTTTCGTTCC	GGCATTGCTCTGCCCATAGATG
TGFB2	AAGAAGCGTGCTTTGGATGCGG	ATGCTCCAGCACAGAAGTTGGC
CYR61	CAGGACTGTGAAGATGCGGT	GCCTGTAGAAGGGAAACGCT
BIRC5	ATCGCCACCTTCAAGAACTG	GGCCAAATCAGGCTCGTTCT
AXL	AACCTTCAACTCCTGCCTTCTCG	CAGCTTCTCCTTCAGCTCTTCAC
AMOTL1	AGTGAGCGACAAACAGCAGACG	ATCTCTGCTCCCGTGTTTGGCA
TEAD4	GAAGGTCTGCTCTTTCGGCAAG	GAGGTGCTTGAGCTTGTGGATG
GAPDH	GTCTCCTCTGACTTCAACAGCG	ACCACCCTGTTGCTGTAGCCAA

All the siRNAs and primers used in the study were synthesized by Genepharma.

Table S3: List of IC50 value in Figure S2.

Cell Line	IC50(μM)									
	VS-4718	IN10018	Crizotinib			Entrectinib				
			DMSO	+VS-4718 3μM	+VS-4718 5μM	DMSO	+IN10018 3μM	+IN10018 5μM	+VS-4718 3μM	+VS-4718 5μM
SNU-668	14.84	12.30	7.6	4.372	1.947	10.30	6.371	4.734	9.031	5.293
NUGC-4	18.97	12.33	11.48	8.20	5.785	17.72	11.83	9.122	14.25	8.858
MGC-803	17.03	10.38	10.41	6.988	3.997	6.717	4.259	1.681	4.419	2.906
MDA-MB-231	15.59	20.09	8.28	6.125	3.889	5.9	2.667	1.747	3.434	2.45
Hs-578t	19.31	14.52	6.906	5.321	3.532	11.4	6.475	3.504	7.21	5.9
